# Mathematical Modeling in Drug Metabolism and Pharmacokinetics: Correct In Vitro, Not Always Valid In Vivo

**DOI:** 10.3390/ph19010160

**Published:** 2026-01-15

**Authors:** Leslie Z. Benet, Jasleen K. Sodhi

**Affiliations:** Department of Bioengineering and Therapeutic Sciences, Schools of Pharmacy and Medicine, University of California San Francisco, San Francisco, CA 94143, USA

**Keywords:** Kirchhoff’s laws, differential equations, drug metabolism, pharmacokinetics, hepatic clearance, renal clearance, mean residence times, bioavailability

## Abstract

**Background/Objectives**: Chemical and metabolic kinetics have historically been derived from mass balance differential equations expressed in terms of amounts, and this framework was later extended to pharmacokinetics by converting amount-based equations to concentration-based clearance relationships. That conversion is valid for fixed-volume in vitro experiments, but may be unreliable in vivo, where input, distribution, and elimination can occur in different volumes of distribution. The objective of this study is to present an alternate, mechanistically agnostic framework for deriving pharmacokinetic relationships by adapting Kirchhoff’s Laws to treat pharmacokinetic systems as networks of parallel and in-series rate-defining processes, and to identify where differential equation approaches fail in vivo. **Methods**: Clearance and rate constant equations were derived using the adapted Kirchhoff’s Laws by summing parallel rate-defining processes and summing inverses for in-series processes, explicitly incorporating organ blood flow, net transporter, and delivery site effects. The resulting expressions were compared with differential equation hepatic disposition elimination models (well-stirred, parallel tube, dispersion) and the Extended Clearance Concept (ECC). Mean residence time concepts were used to extend the framework to oral input, and the full approach was applied to a case study of a hypothetical drug (KL25A). **Results**: The adapted Kirchhoff-based approach reproduced standard pharmacokinetic analyses without mechanistic organ assumptions and yielded model-independent hepatic and renal clearance equations that include blood flow, net transport, and delivery kinetics. Inconsistencies with the traditional differential-based derivations were highlighted, including the interpretation of pharmacokinetics associated with slow absorption site clearance, as illustrated by KL25A. **Conclusions**: For linear drug metabolism and pharmacokinetics, clearance and rate constant relationships can be derived by summing parallel and in-series rate-defining processes, without differential equations. Differential equation methods may misestimate in vivo clearance and bioavailability when drug input is slow or when volumes of distribution differ across processes. The adapted Kirchhoff framework offers a simpler, model-independent basis for interpreting clinical data.

## 1. Introduction

Chemical (metabolism) kinetics have been defined in terms of differential equations since the earliest quantitative study by Wilhelmy [1] in 1850 on the acid-catalyzed conversion of sucrose, and the studies of Harcourt [2] in 1857 who first conducted experiments to follow the course of a chemical change. This differential equation approach also includes the transformational enzyme kinetic studies of Michaelis and Menten [3] and the translation of their work to a straight-forward, teachable differential equation derivation by Briggs and Haldane [4]. Mathematically, the differential equations are expressed in terms of rate constants and amounts/concentrations. They are then solved either by setting the differential rate term to zero under steady-state conditions, or by integrating the differential equation over all time to yield the relationship between measured amounts/concentrations and the rate constants. This general procedure has been followed for more than 150 years. Here, we show that although this differential equation approach allows determination of the rate constants, the same results can be simply obtained using adapted Kirchhoff’s Laws from physics, without deriving the relationships from differential equations. More importantly, the application of the differential equation approach to pharmacokinetic analysis over the past 50 years to determine in vivo drug clearance may yield invalid results in many cases, but not when drug clearance is measured following an iv bolus dose for a drug following linear kinetics.

We recently demonstrated in a series of 11 papers—beginning with our 2022 Pharmacology and Therapeutics publication [5], culminating in our recent 2025 Pharmacological Reviews manuscript [6], and further supported by a 2025 tutorial aimed at simplifying pharmacokinetics for drug dosing decisions in clinical medicine [7]—that clearance equations for linear processes in pharmacokinetics can be simply derived without relying on differential equations. Our initial presentation described the approach as an adaptation of Kirchhoff’s Laws from physics [8,9], and we now recognize that mean residence time concepts [10] yield the same outcome, independent of any electrical circuitry analogy. Here, we use this new methodology to derive the rate constant and clearance equations for first order processes in drug metabolism and pharmacokinetics. We do not address its application to saturable processes, as this will be more fully covered in a subsequent paper.

In 2022, we recognized that determining rate constants and clearance for both in vitro and in vivo pharmacological processes [5] is based on the principle that when two or more rate-defining processes occur in parallel, the total measured rate constant or clearance equals the sum of those rate-defining processes. Conversely, when two or more rate-defining processes are in series, the inverse of the total measured rate constant or clearance equals the sum of the inverse of those rate-defining processes. These relationships are given in Equations (1) and (2) for rate constants.

(1) $$k_{total}=k_{rate-defining\;parallel\;process\;1}+k_{rate-defining\;parallel\;process\;2}+\ldots .\;\;\;\;\;\;$$

(2) $$\frac{1}{k_{total}}=\frac{1}{k_{rate-defining\;in\;series\;process\;1}}+\frac{1}{k_{rate-defining\;in\;series\;process\;2}}+\ldots .\;\;$$

and Equations (3) and (4) when applied to clearance:

(3) $${CL}_{total}={CL}_{rate-defining\;parallel\;process\;1}+{CL}_{rate-defining\;parallel\;process\;2}+\ldots .\;\;\;\;\;$$

(4) $$\frac{1}{{CL}_{total}}=\frac{1}{{CL}_{rate-defining\;in\;series\;process\;1}}+\frac{1}{{CL}_{rate-defining\;in\;series\;process\;2}}+\ldots .$$

A rate-defining process is one that, on its own, could potentially define a total rate constant or total clearance, one that is possible to measure experimentally when it solely determines the rate constant or clearance, and for in-series processes is a positive value. For example, for a series of drug metabolism steps, if the first step is significantly slower than the subsequent metabolic steps, the rate constant of that first step will govern the time-course of both the metabolism of parent compound and formation of all subsequent metabolites. Similarly, if delivery of the parent drug molecule into the system is extremely slow, the rate constant for delivery will define the entire process, including all subsequent steps. If hepatic drug clearance is extremely fast, the overall clearance of the drug will be determined by the rate of delivery to the liver, i.e., hepatic blood flow. Based on Equations (1)–(4), the total rate constant or the total clearance can be simply derived by summing parallel processes, and the inverse of the total average rate constant or clearance can be derived by summing the inverse of processes in series. The definition of parallel and in-series processes is not related to positional characteristics, but rather to whether the value of one process affects the value of other processes. A parallel process does not affect the value of any other parallel process. In-series processes follow one another, with the value of each process being affected by the preceding one. For example, if a compound is eliminated by two different processes that do not affect one another, these are parallel processes because the rate constant or clearance for process 1 does not affect the rate constant or clearance for process 2, even if positionally process 1 is proximal to process 2 (e.g., elimination of a drug in the liver by metabolism and biliary excretion). However, if the compound is eliminated by two sequential processes such as a resultant metabolite formed by process 1 being further eliminated by process 2, these are in-series processes, with the values of the rate constant describing process 1 affecting the value of the measured rate constant defining process 2 (e.g., a drug metabolized in the liver and the resulting metabolite excreted via the bile or urine).

The application of the adapted Kirchhoff’s Laws to rate constants is similar to that described by Cleland [11] for steady-state solutions. However, Cleland and others summarizing his approach do not teach that this application is limited to rate-defining processes, nor do they recognize the relevance of clearance, as their applications only apply to amounts, not concentrations, as the driving force for elimination. As a result, these approaches are not directly applicable to in vivo kinetics.

In the present work, we review our previous approaches represented by the 11 peer-reviewed publications that describe and justify this new approach to derive total clearance and total rate constant relationships independent of differential equations but concentrate here in more detail to specific mathematical modeling applications that can lead to incorrect analyses for in vivo data.

## 2. Materials and Methods

### 2.1. Application to First Order Processes as a Demonstration

**Scheme 1:**
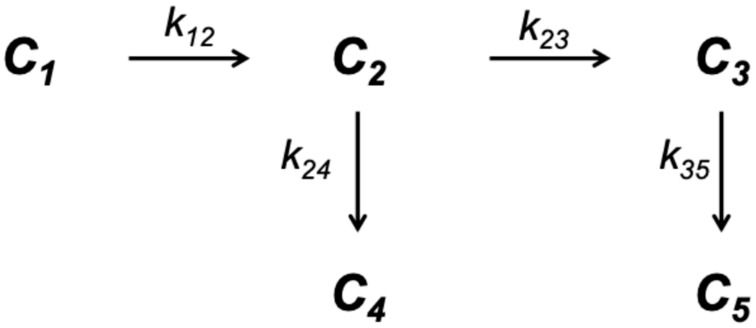
Compound C_1_ that reacts to give four other compounds by first order processes as per the designated rate constants.

A first order chemical reaction sequence is described in Scheme 1 containing both parallel (k_23_ and k_24_) and in-series (k_12_ and k_35_) rate-defining processes.

All compounds are available and the rate of elimination of each can be measured giving the four individual rate constants (*k_12_* = 0.06 h^−1^; *k_23_* = 0.15 h^−1^; *k_24_* = 0.20 h^−1^; *k_35_* = 0.50 h^−1^) for an initial amount of Compound **1** ($C_{1}^{0}=300\;mg$), allowing demonstration of the advantages of adapted Kirchhoff’s Laws for linear systems. For this demonstration we ask, what is the measured average rate constant describing the elimination rate of Compound **3** (C_3_) as a function of time (which is the inverse of the mean residence time of Compound **3**)? Employing the traditional differential equation approach commonly utilized in drug metabolism, chemical kinetics, and pharmacokinetics, one must derive the exponential equation that describes the time course of Compound **3**. Doing so requires solving three coupled differential equations, i.e., $\frac{dC_{3}}{dt},\;\frac{dC_{2}}{dt},\;and\;\frac{dC_{1}}{dt}.\;$Using LaPlace transforms and input and disposition functions [12], the following equation would be obtained:

(5) $$C_{3}=\frac{k_{12}\cdot k_{23}\cdot C_{1}^{0}}{\left(k_{23}+k_{24}-k_{12}\right)\cdot \left(k_{35}-k_{12}\right)}e^{-k_{12}\cdot t}\;+\frac{k_{12}\cdot k_{23}\cdot C_{1}^{0}}{\left(k_{12}-k_{23}-k_{24}\right)\cdot \left(k_{35}-k_{23}-k_{24}\right)}e^{-\left(k_{23}+k_{24}\right)\cdot t}\;+\frac{k_{12}\cdot k_{23}\cdot C_{1}^{0}}{(k_{12}-k_{35})\cdot (k_{23}+k_{24}-k_{35})\cdot }e^{-k_{35}\cdot t}$$

Since the individual parameters have known values:

(6) $$C_{3}=21.16e^{-0.06\;t}-62.07e^{-0.35\;t}+40.9{1e}^{-0.50\;t}$$

One may now calculate the mean residence time for C_3_, by determining area under the moment curve (AUMC) as the sum of the coefficients divided by the exponents squared, and area under the curve (AUC) as the sum of the coefficients divided by the exponents [10].

$$\frac{{AUMC}_{C_{3}}}{{AUC}_{C_{3}}}=\frac{\frac{21.16}{0.06^{2}\;}-\;\frac{62.07}{0.35^{2}}+\frac{40.91}{0.50^{2}}}{\frac{21.16}{0.06}-\frac{62.07}{0.35}+\frac{40.91}{0.50}}=\frac{5535}{257}=21.5\;h$$

Thus, even though the mean residence time for compound C_3_ would be less than 1.5 h (calculated as 1/k_35_) when only the direct elimination of C_3_ itself was measured, the mean residence time of C_3_ is more than 15-fold longer when considering its reaction rate starting from compound C_1_.

Alternatively, one may more simply solve for the mean residence time for C_3_ using adapted Kirchhoff’s Laws. There are three rate-defining processes in series affecting the measured C_3_ amount (one of which involves a sum of parallel processes). Therefore,

$${MRT}_{C_{3}}=\frac{1}{C_{3}\;measured\;average\;elimination\;rate\;constant}=\frac{1}{k_{12}}+\frac{1}{k_{23}+k_{24}}+\frac{1}{k_{35}}$$

since the inverse of the average elimination rate constant is the mean residence time (*MRT*). Substituting the given values for the parameters

$${MRT}_{C_{3}}=\frac{1}{C_{3}\;measured\;average\;elimination\;rate\;constant}=\frac{1}{0.06}+\frac{1}{0.15+0.20}+\frac{1}{0.50}=21.5\;h$$

As noted above, ${MRT}_{C_{3}}$ (when the reaction is started from C_1_) is simply the inverse of the measured average elimination rate constant for C_3_. By using the adapted Kirchhoff’s Laws, the complex differential equation derivation steps become unnecessary. Thus, although traditional differential equation methods will correctly determine the amount–time relationships for this first order system, the adapted Kirchhoff’s Laws approach greatly simplifies the derivation process and eliminates the need for complex differential equation derivations. That is, the adapted Kirchhoff’s Laws approach can transform the analysis of complex processes into a simple additive relationship.

**Scheme 2:**
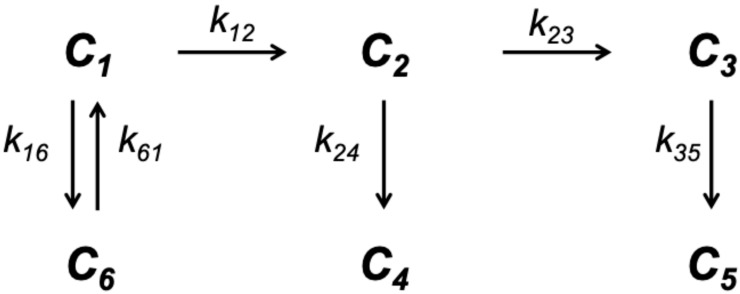
The addition of a reversible metabolic or distribution process affecting C_1_ in Scheme 1.

As noted at the end of the Introduction, this application is identical to that of the simplification of Cleland [11] for derivations of rate constants and amounts. However, consider Scheme 2, where the process depicted in Scheme 1 now includes reversible rate constants that represent drug C_1_ going to and back from C_6_.

No matter what the values of *k_16_* and *k_61_* are, ${MRT}_{C_{3}}$ will always be 21.5 h, since *k_16_* and *k_61_* are not rate-defining processes, a topic not addressed by Cleland [11]. For an in vitro reaction, *k_16_* and *k_61_* could reflect the time course of a reversible metabolite C_6_, which is not eliminated. As C_1_ is converted to C_2_, C_6_ can revert back to C_1_. However, for an in vivo pharmacokinetic process, k_16_ and k_61_ more likely reflect the distribution seen for all drugs into hypothetical body spaces that are not in instantaneous equilibrium with the systemic circulation (i.e., as in compartmental modeling). For in vivo processes, clearance is the relevant measure of elimination, not a rate constant. As we have reported [6,13], it is not possible to convert a rate constant equation derived from differential equations (Equations (1) and (2)) into an in vivo clearance equation, except when clearance is measured following iv bolus dosing. This is because for in vivo conditions, the different driving force processes are associated with different volumes of distribution, and so converting amounts to concentrations using a single systemic volume of distribution value is incorrect. Consider Scheme 2 as an in vitro study where C_1_ is metabolized to C_2_→C_6_. Here, both the rate constant characterizing the elimination of C_3_ and the clearance of C_3_ following addition of C_1_ to the reaction vessel may be determined, since all of the reactions occur in the same fixed volume of fluid within the reaction vessel. For Scheme 1 and Scheme 2 under in vivo conditions, it is also possible to characterize the rate constant characterizing the elimination of C_3_ following dosing of C_1_, but it is not possible to always correctly derive the clearance of C_3_ following dosing of C_1_ using differential equations since each of the six compounds will have a different volume of distribution, and in converting a differential amount equation to a concentration equation, only one volume term can be inserted into the equation [6,13]. Thus, deriving Equations (3) and (4) based on the adapted Kirchhoff’s Laws allows linear clearance parameters to be derived independent of differential equations and without requiring steady-state or pseudo-steady-state assumptions for their solution.

Our definition of rate-defining processes is critical in utilizing this novel approach to deriving total rate constants and total clearance relationships. That is, a rate-defining process is one that, on its own, could potentially define a total rate constant or total clearance and one that is possible to measure experimentally when it solely determines the rate constant or clearance. Thus, both in Scheme 1 and Scheme 2, *k_23_* and *k_24_* must be added since measuring the elimination of C_2_ only is a function of both of these rate constants and if elimination of C_2_ is the slowest step, the measured rate will be a function of the sum of these rate constants. In Scheme 2, *k_16_* and *k_61_* are not rate-defining processes. These rate constants and their ratio can never be measured experimentally; they can only be calculated making model assumptions and elimination of C_1_, C_2_, and C_3_ will not be affected by the values of *k_16_* and *k_61_*.

### 2.2. The Universal Relationship to Simply Derive Total Rate Constant and Clearance Equations

A very simple general approach [6,7,13,14] can be utilized to derive all clinically relevant total rate constant and clearance relationships as given in Equations (7) and (8).

(7) $$\frac{1}{k_{total}}=\frac{1}{k_{rate-defining\;entering\;processed}}+\frac{1}{k_{rate-defining\;leaving\;processes}}$$

(8) $$\frac{1}{{CL}_{total}}=\frac{1}{{CL}_{rate-defining\;entering\;processed}}+\frac{1}{{CL}_{rate-defining\;leaving\;processes}}$$

where the rate constant and clearance entering processes are those that deliver the drug to the site of elimination (e.g., systemic circulation), while the rate constant and clearance leaving processes are those that eliminate the drug from the systemic circulation. Now, we utilize Equation (8) to characterize the clinically relevant characteristics for hepatic and renal clearance, and Equations (7) and (8) to characterize the terminal rate constant and clearance for drug exhibiting “flip-flop” kinetics [15], where the rate of input is slower than the rate of elimination.

## 3. Deriving Renal and Hepatic Clearance

### 3.1. Renal Clearance

When deriving renal clearance (*CL_R_*) following an iv bolus dose, *CL_entering_* is kidney blood flow (*Q_R_*), while *CL_leaving_* is the sum of two parallel processes, glomerular filtration (*CL_filtration_*) and the net transporter-mediated processes, i.e., the difference between renal transporter-related secretion (*CL_R,sec_*) and renal reabsorption (*CL_R,reab_*). These processes are parallel because filtration clearance has no effect of transporter clearance, and vice versa, as given in Equation (9).

(9) $$\frac{1}{{CL}_{R}}=\frac{1}{Q_{R}}+\frac{1}{f_{uB}\cdot GFR+({CL}_{sec}-{CL}_{reab})}$$

As we previously noted [7], prior to our introduction of this relationship, Equation (9) had not been presented because it cannot be derived using differential equations to incorporate renal blood flow. In 1960, when Bricker et al. [16] introduced the intact nephron hypothesis suggesting that measures of *GFR* alone would predict changes in *CL_R_* (i.e., when *GFR* decreased a comparable decrease in net renal transporter effects and passive permeability would occur), renal blood flow was not considered. It is true that for many renally excreted drugs, renal blood flow will have minimal impact and need not be considered because their clearance is well below renal blood flow value; however, this is not always true. For example, the measured *CL_R_* of metformin is approximately 600 mL/min in healthy young humans, and with negligible plasma protein binding (f_uP_ ≈ 1.0) the *CL_filtration_* = GFR ≈ 120 mL/min. Therefore, previously, the net secretory clearance of metformin (*CL_R,sec_* − *CL_R,reab_*) was assumed to equal to approximately 480 mL/min based on the traditional equations used to estimate renal clearance. However, when kidney blood flow is included (*Q_R_* ≈ 1200 mL/min) as in Equation (9), the secretory clearance is instead found to be 1080 mL/min, a value greater than twofold higher than estimated with the previous approach for renal clearance. This analysis indicates that the field has been significantly underestimating the importance of tubular transport for drugs exhibiting moderate renal clearance values, such as metformin and for 21 other drugs as previously listed [6]. This has significant implications when using in vitro measurements to predict in vivo parameters, i.e., IVIVE (in vitro—in vivo extrapolation). It will also be highly relevant in predicting and defining the extent of transporter drug–drug interactions.

### 3.2. Hepatic Clearance

In deriving hepatic clearance (*CL_H_*) following an iv bolus dose, *CL_entering_* reflects two in-series processes, liver blood flow (*Q_H_*) and the difference between hepatobasolateral membrane influx (*CL_H,influx_*) and hepatobasolateral membrane efflux (*CL_H,efflux_*), while *CL_leaving_* is the sum of two parallel processes, hepatic metabolism (*CL_H,met_*) and biliary excretion (*CL_H,bil_*), as given in Equation (10).

(10) $$\frac{1}{{CL}_{H}}=\frac{1}{Q_{H}}+\frac{1}{\left({CL}_{H,influx}-{CL}_{H,efflux}\right)}+\frac{1}{\left({CL}_{H,met}+{CL}_{H,bil}\right)}$$

For hepatic clearance, the effects of protein binding must also be considered by inserting fraction unbound in blood (*f_u,B_*) and intrinsic (*int*) clearances (i.e., clearances independent of blood flow and protein binding), thereby converting Equation (10) to Equation (11), where *CL_H,int_* is the sum of the intrinsic metabolic and biliary clearances.

(11) $$\frac{1}{{CL}_{H}}=\frac{1}{Q_{H}}+\frac{1}{f_{uB}\cdot ({CL}_{H,int,influx}-{CL}_{H,int,efflux})}+\frac{1}{f_{uB}\cdot {CL}_{H,int}}$$

Now, we have simply derived the renal and hepatic clearance equations by applying parallel and in-series rate-defining processes independent of differential equations.

#### 3.2.1. When Hepatocellular Transport Is Not a Rate-Defining Process

When hepatocellular transport is not a rate-defining process (i.e., when the second term on the right-hand sides of Equations (10) and (11) is omitted), solving Equation (11) yields the following:

(12) $${CL}_{H}=Q_{H}\cdot \frac{f_{uB}\cdot {CL}_{H,int}}{Q_{H}\cdot f_{uB}\cdot {CL}_{H,int}}$$

which is a very familiar equation that has been considered to be the well-stirred model (WSM) equation for the past half-century [17,18]. Equation (12) was derived here based on the adapted Kirchhoff’s Laws, making no assumptions related to mechanistic characteristics of hepatic elimination. Equation (12) is organ model-independent, and we maintain that it is the general relationship between *Q_H_*, *f_u,B_*, and *CL_int_* when only systemic concentrations can be measured. “When only systemic concentrations can be measured” is the critical condition.

As we have previously written [6], “The mechanistic model independence of Equation (12) explains why all published quality experimental data in the isolated perfused rat liver (IPRL) align exclusively with this equation, as we reported [19]. By quality data, we refer to data that are robust and reliable, particularly in studies where experimental inconsistencies have been revealed upon closer examination. IPRL steady-state studies are the only experimental outcomes that can differentiate the WSM from the parallel tube model (PTM) and the infinite number of intermediary dispersion models (DMs), which depend on the dispersion coefficient assumed. Our review of published IPRL studies supports the generalizability of Equation (12) over the alternatively suggested hepatic disposition models [19], by critically examining IPRL data in four studies with high-clearance drugs (lidocaine, meperidine, and propranolol), four studies with high-clearance compounds (galactose and taurocholate), and five studies where two low-clearance drugs (diazepam and diclofenac) were made high clearance by manipulating protein binding (as detailed in Table 1 of ‘Summary of IPRL studies for high-clearance substances’ in Sodhi et al. [19]). We were particularly struck by the fact that the highest cited paper supporting the PTM [20] reported several findings that contradict hepatic physiology, including that increasing hepatic blood flow paradoxically led to decreased hepatic clearance for the high extraction ratio compound galactose in 4 of the 10 studies analyzed. This underscores the importance of using well founded data to support or challenge pharmacokinetic theory”.

#### 3.2.2. When Hepatocellular Transport Is Clinically Relevant

When hepatocellular transport is relevant and affecting *CL_H_*, all three parameters on the right-hand sides of Equations (10) and (11) must be considered. However, in practice, the relationship is most often evaluated under conditions where *Q_H_* is much greater than both the second term (the net transport) and the third term (elimination clearances) on the right-hand side of Equation (11). Then

(13) $${CL}_{H}=\frac{f_{uB}\cdot {CL}_{H,int}\cdot ({CL}_{H,int,influx}-{CL}_{H,int,efflux})}{{CL}_{H,int}+({CL}_{H,int,influx}-{CL}_{H,int,efflux})}=\frac{f_{uB}\cdot {CL}_{H,int}}{1+\frac{{CL}_{H,int}}{\left({CL}_{H,int,influx}-{CL}_{H,int,efflux}\right)}}$$

In Equation (13), when ${CL}_{H,int}\;$is much greater than $\left({CL}_{H,int,influx}-{CL}_{H,int,efflux}\right),$ hepatic basolateral transport is the rate-limiting step, while when $\left({CL}_{H,int,influx}-{CL}_{H,int,efflux}\right)$ is much greater than ${CL}_{H,int}$, then hepatic elimination is the rate-limiting step. However, Equation (13) also describes the *CL_H_* relationship for all intermediate characteristics of hepatic clearance between these two boundary conditions.

#### 3.2.3. Net Hepatic Basolateral Transport Processes

One will immediately notice that in our derivation of hepatic clearance using the adapted Kirchhoff’s Laws approach of summing parallel and in-series processes (Equations (10)–(13)) that we include the net hepatic basolateral transporter process (i.e., the difference between influx and efflux), just as investigators have long considered the net secretory and reabsorptive processes in the kidney in Equation (9) when defining renal clearance. This approach differs from the widely employed Extended Clearance Concept (ECC), where influx and efflux hepatic transporter clearances are included as separate parameters, as discussed in Section 3.2.5 below. Very recently, we have addressed this topic in detail [14].

First, there may be concern about the relevance of the net influx and efflux clearances term in Equations (10) and (11) for hepatic clearance when efflux is greater than influx. Historically, analogous negative values for the net secretory-reabsorption clearance term for renal processes in Equation (9) have been readily interpretable by the field. Namely, this occurs for a number of drugs for which *CL_R,reab_* is greater than *CL_R,sec_*, yielding a measured *CL_R_* that is less than *CL_R,filtration_*. However, for the liver, within the adapted Kirchhoff’s Laws framework, the definition of a rate-defining process (as outlined in the Introduction) is key to correctly interpreting a potentially negative net hepatic basolateral transporter clearance, as a negative net clearance terms cannot be rate-defining. For the liver, it is certainly possible that *CL_H_*_,*int,efflux*_ can be greater than *CL_H_*_,*int,influx*_, which would yield a negative net value. However, a negative net transporter clearance cannot be rate-defining, just as passive permeability clearances of drug passing back and forth between peripheral compartments cannot be rate-defining and thus do not appear in the clearance equations. As given in the definition of rate-defining processes, the process must be clinically measurable under some conditions, and, in practice, an overall negative clearance cannot be measured. Importantly, many processes can occur in vivo that are not rate-defining and thus do not appear in the clearance equations. In summary for this first point, although negative clearance values can be physiologically meaningful in certain contexts such as renal reabsorption, the structure of hepatic clearance pathways prevents negative basolateral transporter clearances from being rate-defining, ensuring that only clinically measurable, rate-defining processes appear in clearance equations.

Second, based on the differential equation derivation of the ECC as detailed in the next section, it is proposed that permeability clearances for hepatobasolateral transporter-mediated influx can be experimentally determined in the same way that one determines passive permeability values, i.e., by measuring initial entry into the hepatocyte or the hepatocellular membrane under conditions where concentrations driving efflux are minimal and can be ignored. However, as we very recently discussed [14], “transporters function either to enhance the absorption and distribution of beneficial compounds, or to serve as a protective mechanism in limiting the entry of xenobiotics into the body and specific organs. Transporters would not adequately serve this latter function if they only effluxed drug from outside of the membrane. Thus, attempts to quantitatively determine directional transporter clearances based on initial rates of membrane passage are not valid. Measurements of initial rates of transporter clearance, whether for influx and efflux, reflect the net difference between influx and efflux clearances, as transport across a membrane inherently involves a balance between opposing fluxes. Since concentrations within the membrane cannot be directly measured, it is not possible to separate the influx from efflux components”.

#### 3.2.4. There Is No Clinical Relevance to Mechanistic Models of Hepatic Elimination

The initial proposed mass balance derivation for the WSM [17], which the first author of this manuscript co-authored, assumed the steady-state relationship in Equation (14):

(14) $${CL}_{H,WSM}\cdot C_{Blood}=Q_{H}\cdot f_{uB}\cdot \left(C_{in,u}-C_{out,u}\right)={CL}_{H,int}\cdot C_{H,u}={CL}_{H.int}\cdot C_{out,u}$$

where *C_Blood_* is the steady-state concentration of total drug in the blood, *C_in,u_* and *C_out,u_* are the steady-state unbound concentrations of drug in blood entering and leaving the liver, respectively, and *C_H,u_* is the average concentration of unbound drug within the liver. In the WSM, *C_H,u_* is assumed to be equal to the unbound drug concentration in the blood exiting the liver, *C_out,u_*, because the organ is assumed to be infinitely mixed. In 2018, we first began to question the validity of Equation (14) [21], asking why the elimination of the drug from the systemic circulation is a function of hepatic blood flow, whereas elimination from the liver is not a function of hepatic blood flow, as given in Equation (14a):

(14a) $${CL}_{H,WSM}=\frac{{CL}_{H.int}\cdot C_{out,u}}{C_{Blood}}$$

If blood clearance is rate-limited by hepatic blood flow, why is liver clearance also not rate limited by hepatic blood flow?

As reviewed by Li and Jusko [22], variants of Equation (14a) form the basis for all the mechanistic models of hepatic elimination (i.e., WSM, PTM and DM), as given in Equation (14b):

(14b) $${CL}_{H\;(WSM,PTM,DM)}\cdot C_{Blood}={CL}_{int,WSM}\cdot C_{out,u}={CL}_{int,PTM}\cdot \frac{C_{in,u}-C_{out,u}}{ln\frac{C_{in,u}}{C_{out,u}}}={CL}_{int,DM}\cdot C_{avg,u,DM}$$

That is, for the three mechanistic models of hepatic elimination used in pharmacokinetic evaluations and in making physiologic-based pharmacokinetic (PBPK) models, it is assumed that the product of the measured steady-state systemic clearance and the steady-state drug concentration measured in blood (left hand side of Equation (14b)) is equal to the product of the average liver concentration at steady-state for that specific model and the intrinsic hepatic clearance for that model, with no direct relationship to liver blood flow. Above in Section 3.2.1 we reviewed the finding that no quality experimental IPRL data support the PTM and DM models; here, we further show that the derivations of those hepatic disposition models are also deficient. As reported above, Equation (12) is derived independent of any mechanistic model based on the adapted Kirchhoff’s Laws approach and defines hepatic clearance when hepatobasolateral transporter effects are not clinically significant.

#### 3.2.5. The Deficiencies of the Extended Clearance Concept

Quantitation of in vivo hepatic transporter effects was presented by Siriani and Pang [23] based on differential equations. Their approach later led to the ECC, as we have reviewed [24], yielding calculation of hepatic clearance via the ECC, *CL_H,ECC_*, as given in Equation (15):

(15) $${CL}_{H,ECC}=\frac{Q_{H}\cdot {CL}_{H,int,influx}\cdot f_{uB}\cdot {CL}_{H,int}}{{CL}_{H,int,influx}\cdot f_{uB}\cdot {CL}_{H,int}+Q_{H}\cdot {CL}_{H,int}+{Q_{H}\cdot CL}_{H,int,efflux}}$$

When *Q_H_* is much greater than the three intrinsic clearances, Equation (15) simplifies to Equation (16):

(16) $${CL}_{H,ECC}=\frac{{CL}_{H,int,influx}\cdot f_{uB}\cdot {CL}_{H,int}}{{CL}_{H,int}+{CL}_{H,int,efflux}}$$

We recently detailed five deficiencies of the ECC [14,25], as listed here:

(a) In Equation (16), for hepatic basolateral transport *CL_H,int,influx_* to be rate-limiting, *CL_H,int,efflux_* must be assumed to be zero or negligible relative to *CL_H,int_*, such that *CL_H,int_* cancels out in the numerator and denominator. Then, *CL_H_*_,*ECC*_ = *f_uB_* · *CL_H_*_,*int,influx*_ and the uptake process is the rate-limiting step in clearance. But why must efflux transport be zero or negligible compared to *CL_H,int_* for hepatobasolateral transport to be rate- limiting? Instead, under the adapted Kirchhoff’s Law framework (Equation (13)), if the net hepatic basolateral flux (i.e., the positive difference between influx and efflux) is much smaller than *CL_H,int_*, then *CL_H_* = *f_uB_* · (*CL_H_*_,*int,influx*_ − *CL_H_*_,int,*efflux*_) and net hepatobasolateral transport becomes rate-limiting.
(b) In the ECC Equation (16), it is not possible for *CL_H,int_* to be rate-limiting unless it is assumed that basolateral influx and efflux are equal, and greater than *CL_H,int_*. That is, in the denominator *CL_H,int_* < < *CL_H,int,efflux_* and then if *CL_H,int,influx_* = *CL_H,int_*_,efflux_ the expression can result in *CL_H_*_,*ECC*_ = *f_uB_* · *CL_H_*_,*int*_. However, if influx equals efflux, this represents a passive process, and the transporter clearances should not be included in the derivation. Instead, using the adapted Kirchhoff’s Law framework (Equation (13)), *CL_H,int_* will be rate-limiting as long as it is significantly smaller than (*CL_H_*_,*int*,*influx*_ − *CL_H_*_,*int,efflux*_).
(c) Equation (16) was derived [23] based on the WSM hypothesis, where overall clearance within the liver is incorrectly assumed to be *f_uB_·CL_H,int_*, a relationship that is independent of hepatic blood flow. We have previously described the basis for this error in detail [6,26] and in Section 3.2.4 above.
(d) Determining *Kp_uu_*, the ratio of the WSM-hypothesized average unbound liver concentration to the measured unbound systemic blood concentration, results in ECC values that are surprisingly always less than unity and lower than hepatic bioavailability *F_H_* (i.e., $1\;-\;\frac{{CL}_{H}}{Q_{H}}$), as we first reported [27]. We consider this to be one of the key reasons to question the validity of the derivation of the WSM and ECC, and this finding remains unchallenged in the published literature.
(e) When measuring only systemic concentrations to determine hepatic clearance, why should hepatic organ clearance not follow the same approach as kidney organ clearance, where, as shown in Equation (9), the two membrane passage parameters are evaluated as a net difference?

We further pointed out [14] that reported outcomes of *CL_H,ECC_* being consistent with rate-limitation by hepatobasolateral transport do not validate Equation (16), since it is experimentally impossible to determine whether the transporter measurement reflects *CL_H,influx_* alone or the net term of (*CL_H,influx_* − *CL_H,efflux_*).

## 4. Drug Input and Mean Residence Time Concepts

Up to this point, we have only considered iv bolus dosing, demonstrating how the adapted Kirchhoff’s Laws approach allows simple derivations of clearance and total rate constants independent of differential equations and any mechanistic assumptions, through the correct summation of parallel and in-series rate-defining processes. We also have shown that commonly used mechanistic models of hepatic elimination are not supported by experimental data and emphasized the need to incorporate organ blood flow and net transporter effects in analyzing clinical data. However, the deficiencies alone do not by themselves demonstrate our central claim as stated in title of this manuscript—namely, that presently used mathematical methodologies in drug metabolism and pharmacokinetics may not provide valid in vivo analyses. This is addressed subsequently, by extending the iv bolus framework developed above as the foundation in analyzing alternate routes of drug input.

### 4.1. Mean Residence Times

Mean residence time (*MRT*, i.e., the average time a drug is measurable in the systemic circulation) concepts allow a simple way to analyze clinical data [10] following alternate routes of drug administration by including mean absorption time (*MAT*).

(17) $${MRT}_{system\;oral\;dose}={MRT}_{iv\;bolus}+MAT$$

For first order systems, *MRT* is the reciprocal of the first order rate constants describing the process. Thus,

(18) $$\frac{1}{k_{system\;oral\;dose}}=\frac{1}{k_{system\;iv\;bolus}}+\frac{1}{k_{absorption}}$$

Equation (18) follows the same mathematical format as the in-series relationship for rate constants in Equation (2). In effect, pharmacokinetic analyses have been using an adapted Kirchhoff’s Laws structure for rate constants since Yamaoka et al. introduced MRT concepts in 1979 [10], even if this was not explicitly recognized at the time. Solving Equation (18) for *k_system oral dose_* gives

(19) $$k_{system\;oral\;dose}=\frac{k_{system\;iv\;bolus}\cdot k_{absorption}}{k_{system\;iv\;bolus}+k_{absorption}}=\frac{k_{system\;iv\;bolus}}{1+\frac{k_{iv\;bolus}}{k_{absorption}}}$$

When *k_absorption_* is significantly greater than *k_system iv bolus_*, the terminal phase of the concentration–time curve will have a slope corresponding to *k_system iv bolus_*., as is typical for rapidly absorbed drugs. In contrast, when absorption is slow—often deliberately for controlled release dosage forms—the terminal slope will reflect *k_absorption_*. Hence, Equation (19) therefore provides a mathematical basis for the flip-flop model [15], not only at the boundary conditions, but across the full range of intermediate absorption–elimination relationships.

### 4.2. Clearance from the Absorption Site (CL_absorption site_) and Absorption Site Volume of Distribution (V_absorption site_)

Although MRT concepts are expressed in terms of rate constants, clinical drug dosing decisions are made based on clearance values. The clearance equivalent of Equation (18) is

(20) $$\frac{1}{{CL}_{system\;oral\;dose}}=\frac{1}{{CL}_{system\;iv\;bolus}}+\frac{1}{{CL}_{absorption\;site}}$$

Clearance from the absorption site is a parameter that had not been explicitly considered prior to Benet et al. [28] but is easily determined following iv bolus and oral dosing from Equation (20). For first order absorption, it equals the product of *k_absorption_* and the absorption site volume of distribution (*V_absorption site_*), a second new pharmacokinetic parameter introduced in that work [28], which can be determined if *k_absorption_* can be determined. *V_absorption site_* is the non-physiological parameter that is defined as the amount of drug at the absorption site divided by the concentration at that site. *V_absorption site_* has no more physiologic relevance than the volume of distribution steady state (*V_ss_*), and will certainly not be equal to *V_ss_*, as we previously described [13,28]. Solving Equation (20) for *CL_system oral dose_* gives

(21) $${CL}_{system\;oral\;dose}=\frac{{CL}_{system\;iv\;bolus}\cdot {CL}_{absorption\;site}}{{CL}_{system\;iv\;bolus}+{CL}_{absorption\;site}}=\frac{{CL}_{system\;iv\;bolus}}{1+\frac{{CL}_{system\;iv\;bolus}}{{CL}_{absorption\;site}}}$$

Thus, the clearance measured following oral dosing will only equal *CL_system iv bolus_* if *CL_absorption site_* is significantly greater than *CL_system iv bolus_*. This will often be true, but drug clearance from the absorption site can also be very slow for several reasons, for example with solubility- or dissolution-limited absorption, low permeability molecules, and often deliberately for controlled release dosage forms. We will return to the implications of Equation (21) after discussing bioavailability.

### 4.3. Bioavailability

Bioavailability can be estimated either by determining dose-corrected *AUC*_0_*_→∞_* measures following oral and iv bolus dosing and/or dose-corrected amounts of unchanged drug excreted in urine (*U_∞_*), by assuming that clearance is unchanged between the iv and oral dosing routes. A key complication in these calculations is that this assumption (i.e., that clearance is the same between dosing routes) may not hold, an issue that was not recognized prior to our work [5,6], showing that clearance can be derived using in-series and parallel rate-defining processes (Equation (21)). If following oral dosing, *CL_absorption site_* is much greater than *CL_system iv bolus_*, then clearance after iv and oral dosing routes are effectively equivalent and the standard bioavailability procedures remain valid. In contrast, if *CL_absorption site_* is not significantly greater than *CL_system iv bolus_*, then Equation (21) indicates that *CL_system oral dose_* will be smaller than *CL_system iv bolus_*. Therefore, in such cases, calculations of bioavailability using systemic concentration measurements may be inaccurate. Conceptually, a slow in-series oral input will have a higher measured systemic *AUC* relative to the same dose administered intravenously, analogous to the earlier example that a very slow metabolic clearance step will rate limit subsequent metabolic steps. As explained in Benet et al. [28] with specific examples provided, slow input from the site of administration can even result in systemic bioavailability values greater than unity, values that are statistically significantly higher than bioavailability values derived from measurement of unchanged drug in the urine. These differences are also subsequently reflected in statistically significant renal clearance values that are a function of route of dosing. As will be demonstrated in subsequent calculations, Equation (21) directly contradicts the long-standing differential equation assumption that the rate of drug input does not affect measured drug clearance.

## 5. Analyzing Pharmacokinetic Data Following Oral and IV Bolus Administration

### 5.1. IV Bolus Dosing

**Figure 1:**
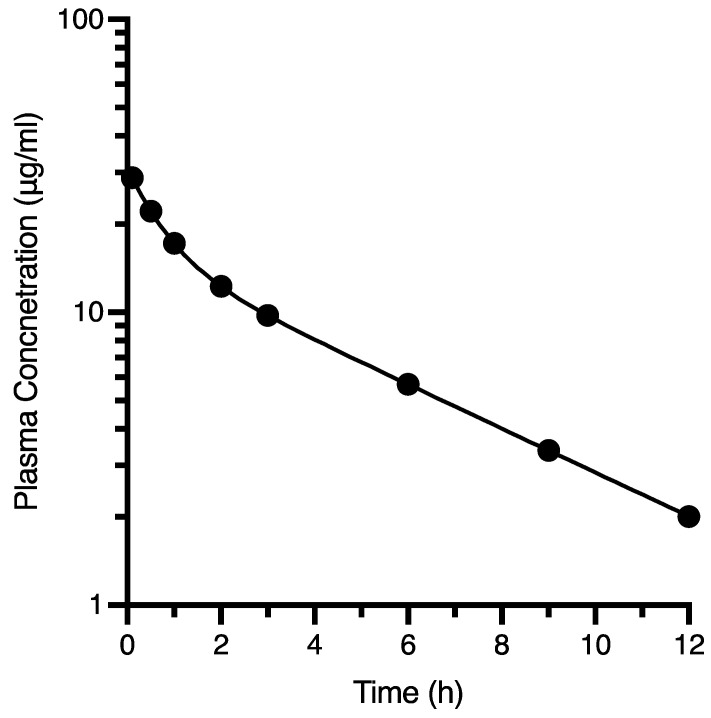
Semilogarithmic plasma concentration–time curve following an intravenous 2500 mg bolus dose of KL25A to a 70 kg man. Data best computer fit to a biexponential equation $C_{P}=15\cdot e^{-1.39t}+16\cdot e^{-0.173t}$.

Having completed derivation of the basic equations using parallel and in-series rate-defining processes, we begin analyzing pharmacokinetic data for a hypothetical drug KL25A, a poorly soluble, neutral molecule that follows linear kinetics, as we previously presented [7]. The logarithmic concentration–time curve is depicted in Figure 1 for a 2500 mg iv bolus dose of KL25A in a healthy volunteer. The plasma data were best fit to a biexponential equation, $C_{P}=15\cdot e^{-1.39t}+16\cdot e^{-0.173t}$.

#### Determine the Pharmacokinetic Characteristics for the IV Bolus Data

For the data in Table 1 following iv bolus dosing, *AUC_0__→∞_* may be calculated as the sum of the coefficients divided by the exponents ($\frac{15}{1.39}+\frac{16}{0.173}=103\frac{\mu g\cdot h}{mL}$) and *AUMC*_0_*_→∞_* may be calculated as the sum of the coefficients divided by the exponents squared ($\frac{15}{1.39^{2}}+\frac{16}{0.173^{2}}=542\frac{\mu g\cdot h^{2}}{mL}$).

Total clearance for KL25A is determined following an iv bolus dose as

$${CL}_{iv\;bolus}=\frac{Dose}{{AUC}_{0\to \infty }}=\frac{2500\;mg}{103\;\frac{mg\cdot h}{L}}=24.3\;\frac{L}{h}$$

Renal clearance is the fraction of the iv bolus dose excreted unchanged multiplied by the total clearance

$${CL}_{R}=\frac{U_{\infty }}{Dose}\cdot {CL}_{total}=\frac{1430\;mg}{2500\;mg}\cdot 24.3\frac{L}{h}=13.9\;\frac{L}{h}$$

and hepatic clearance is assumed to be the total clearance minus the renal clearance, 24.3 − 13.9 = 10.4$\;\frac{L}{h}$.

Mean residence time of the iv bolus dose $=\frac{AUMC}{AUC}=\frac{542}{103}=5.26\;h$ and $V_{ss}=CL\cdot MRT=24.3\cdot 5.26=127.8\;L\;$ [28]. As so little of the dose is excreted as metabolites, KL25A is eliminated by renal and biliary excretion of unchanged drug. Since the renal clearance of KL25A ($13.9\;\frac{L}{h}$) is markedly greater than renal filtration clearance (0.1·120 $\frac{mL}{min}$ = 12 $\frac{mL}{min}$), the drug is a substantial substrate for renal secretory transporters. As it is a neutral compound, it will not be a substrate for hepatobasolateral transporters, which are organic acid transporting polypeptides, but rather a substrate for apical hepatic transporters into the bile.

### 5.2. Oral Dosing

**Figure 2:**
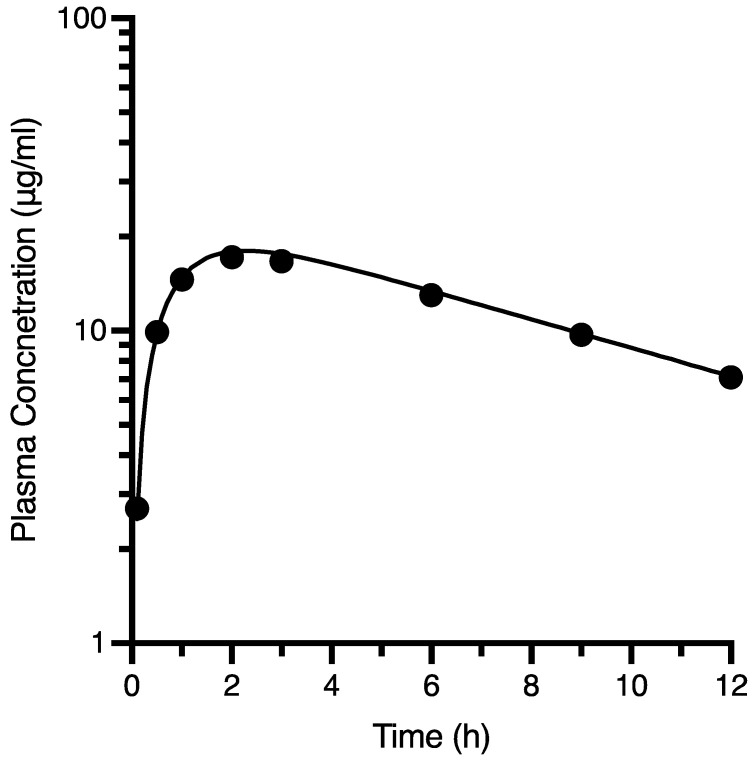
Semilogarithmic plasma concentration–time curve following a 5000 mg oral dose of KL25A to a 70 kg man. Data best computer fit to a biexponential equation $C_{P}=22.7\cdot e^{-0.105t}-22.7\cdot e^{-1.16t}$.

The logarithmic concentration–time curve for a 5000 mg oral dose of KL25A in the same healthy volunteer is depicted in Figure 2. The oral plasma data were also best fit to a biexponential curve:

$$C_{P}=22.7\cdot e^{-0.105t}-22.7\cdot e^{-1.16t},giving\;an\;{AUC}_{0\to \infty }=\frac{22.7}{0.105}-\frac{22.7}{1.16}=197\;\frac{mg\cdot h}{L}$$

#### 5.2.1. Determine the Bioavailability for the Oral Data

From the unchanged urinary amounts

$$F_{urine\;data}=\frac{U_{\infty ,\;oral}}{U_{\infty ,iv\;bolus}}\cdot \frac{{Dose}_{iv}}{{Dose}_{oral}}=\frac{220}{1430}\cdot \frac{2500}{5000}=0.0769$$

From the systemic plasma concentrations

$$F_{plasma\;data}=\frac{{AUC}_{0\to \infty ,\;oral}}{{AUC}_{0\to \infty ,iv\;bolus}}\cdot \frac{{Dose}_{iv}}{{Dose}_{oral}}=\frac{197}{103}\cdot \frac{2500}{5000}=0.956$$

This marked difference in *F* is characteristic of the impact that slow drug delivery clearance has on influencing systemic *AUC* measurements for high-clearance drugs, while having no effect on the actual amount of drug reaching the systemic circulation. There are no assumptions in the urine data, just the doses and measured amounts of unchanged drug in the urine. In contrast, bioavailability measure from systemic concentrations assumes that drug input into the systemic circulation, no matter how slow, has no effect on measured systemic concentrations.

#### 5.2.2. Determine Mean Absorption Time for the Oral Data

From Equation (17)

$$MAT={MRT}_{oral\;dose}-{MRT}_{iv\;bolus}=\frac{\frac{22.7}{0.105^{2}}-\frac{22.7}{1.16^{2}}}{\frac{22.7}{0.105}-\frac{22.7}{1.16}}-5.26=5.13\;h$$

The *MAT* for the oral dose is just slightly less than the total *MRT* of the iv dose. Considering the well-recognized principle that very slow metabolism of a parent drug yields very slow elimination measurements of subsequent metabolites, why have we believed that slow clearance from the drug delivery site would not affect the measured clearance following oral dosing? We will address this in the Discussion (Section 6).

## 6. Discussion

Here, we reviewed our discovery in 2022 [5] and the 10 follow-up publications, demonstrating that all drug metabolism and pharmacokinetic clearance and total rate constant relationships could be derived independent of differential equations using parallel and in-series rate-defining processes adapted from Kirchhoff’s Laws in physics. Our published papers show that the outcomes from these differential equation free relationships provide the same results as many previously published analyses based on differential equation derivations, but with these additions as we previously enumerated [6].

- We explain why all experimental data from steady-state IPRL studies align with what has previously been considered as the WSM of hepatic elimination, a universally recognized unphysiological organ model. None of the published quality experimental data are preferentially consistent with the PTM or DM models of hepatic elimination, although both are considered to be more physiologically relevant than the WSM.
- Since the ECC is based on the WSM differential equation derivation, we demonstrated its limitations, including (i) the fact that the ECC leads to the conclusion that *Kp_uu_* can never be greater than unity and inexplicably reflective of *F_H_*; (ii) the outcome of ECC that hepatobasolateral influx can only be rate-limiting when hepatobasolateral efflux is zero or negligible; (iii) the inability of the ECC equation to explain intrinsic hepatic clearance as rate-limiting unless hepatobasolateral transport is absent; and (iv) the fact that published studies concluding that hepatobasolateral influx rate limits clearance cannot be experimentally distinguished from the adapted Kirchhoff’s Laws derivations showing that net hepatobasolateral transport (influx–efflux) is the rate-limiting condition.
- Explaining why it is possible to observe systemic bioavailability measures that exceed unity for linear systems, and why this is not an experimental error.
- Explaining why statistically significant differences can occur between bioavailability measures derived from systemic concentrations versus measures based on urinary excretion of unchanged drug.
- Explaining why renal clearance can be a function of drug input processes for drugs following linear kinetics.
- Demonstrating how organ blood flow measurements can be simply incorporated in organ clearance derivations.

Using the procedures described, we derived renal and hepatic clearance equations, bioavailability, and mean residence time measures following iv bolus and oral administration for a hydrophilic, neutral, hypothetical drug KL25A following a 2500 mg iv bolus dose and a 5000 mg oral dose in a healthy volunteer.

### 6.1. Lessons from the Pharmacokinetic Analysis and Why the KL25A Demonstration Was Chosen

As we previously reported [7], after recognizing that slow drug input into the systemic circulation can affect measured *AUC*, based on derivation of pharmacokinetic equations using parallel and in-series rate-defining processes adapted from Kirchhoff’s Laws, we presented a hypothetical drug with an intermediate clearance (both by renal and hepatic processes) of 24.3 L/h following iv dosing. However, because of its polarity, KL25A was very poorly absorbed (*F_urine data_* = 7.7%), yet because absorption was so slow, the increase in *AUC* made it appear that oral bioavailability was almost complete (*F_plasma data_* = 95.6%). Since pharmacodynamics is almost always a function of systemic exposure, the magnitude of pharmacodynamic outcome of this oral dose would be expected to be indistinguishable from that of the iv dose, based on the comparable systemic exposures between dosing routes, even in cases of indirect pharmacological mechanisms where the pharmacodynamic time course does not mirror the pharmacokinetic profile. In our recent publication [7], these findings were extended to an example of immediate release (IR) vs. extended release (ER) oral dosage forms (from a publication in preparation).

Lukacsko et al. [29] report a significant decrease in LDL cholesterol and total cholesterol for 149 hypercholesterolemic patients receiving 20 mg daily doses of ER lovastatin vs. 20 mg daily IR lovastatin. Systemic lovastatin concentrations were not measured in this study. However, 2 years earlier, the company reported [30] that daily 20 mg doses of ER lovastatin vs. IR lovastatin, in a different population, gave a 71% average increase in lovastatin *AUC* at day 28. These data are consistent with the KL25A demonstration above, where slow drug clearance from the absorption site leads to a marked increase in *AUC* relative to a comparable iv bolus dose. These findings may lead to a paradigm shift in drug development of new molecular entities, as we recently proposed [31]. Drug developers have often considered molecules with low clearance and longer half-life advantageous, particularly for increasing the likelihood of achieving projected efficacious concentrations and desired pharmacodynamic effects in humans. But then major Pharma is ignoring its greatest strengths, drug development, and expertise in formulation. The KL25A and lovastatin examples presented here suggests that moderate- or high-clearance drugs may be preferable because then systemic clearance can be controlled by the formulation, essentially independent of mechanisms of drug elimination and patient elimination process variability.

### 6.2. Calculation of CL_absorption site_ for KL25A

Wakuda et al. [32] reported calculated values of *CL_absorption site_* for nine drugs in humans where oral and iv doses with measurements of systemic concentrations and urinary elimination of unchanged drug were published. This is accomplished by recognizing that Equation (21) can be extended to include the urinary measure of bioavailability, since there are no measurement assumptions related to this value, as discussed above

(21a) $${CL}_{system\;oral\;dose}=\frac{F_{urine\;data}\cdot {Dose}_{oral}}{{AUC}_{0\to \infty ,oral}}=\frac{{CL}_{system\;iv\;bolus}}{1+\frac{{CL}_{system\;iv\;bolus}}{{CL}_{absorption\;site}}}$$

Therefore, for the data of KL25A,

$${CL}_{system\;oral\;dose}=\frac{F_{urine\;data}\cdot {Dose}_{oral}}{{AUC}_{0\to \infty ,oral}}=\frac{0.0769\cdot 5000}{197}=1.95\;\frac{L}{h}$$

Substituting this value into Equation (21) together with the *CL_system iv bolus_* value of 24.3 $\frac{L}{h}$ leads to *CL_absorption site_* = 2.12 $\frac{L}{h}$. In this hypothetical example, the clearance from the gut is markedly smaller than the iv bolus clearance, yielding this major difference in systemic versus urinary bioavailability measures.

### 6.3. The Reason That Differential Equation Derivations Cannot Explain the KL25A Bioavailability Results

We briefly addressed this topic above at the end of Section 2.1 and in detail for a drug following a single exponential equation when administered as an iv bolus dose in a recent publication [6]. Here we present a general explanation. Historically, as described in the Introduction, rates of drug metabolism were derived using differential equations based on mass balance considerations, that is, the derivations were in terms of amounts and rate constants. The early chemical reactions and, subsequently, in vitro drug metabolism studies were conducted in a beaker with a fixed volume. Thus, measurements of substrates and products were all in the same volume and the amount differential equations could be converted to a concentration differential equation just by dividing the derived amount and rate constant equations by this fixed volume. Therefore, for in vitro measures and for determinations of rates of reaction with respect to amounts in vivo, mass balance is a necessary and sufficient criterion.

However, as detailed above and in our series of papers describing the adapted Kirchhoff’s Laws approach, we have emphasized that in vivo drug dosing decisions and changes in drug dosing clinically are based on changes in drug clearance, not rate constants. Therefore, in pharmacokinetics, mass balance is a necessary but not a sufficient criterion when input or output occurs within a different volume of distribution. In the differential equation derivation for the iv bolus dose of KL25A, the change in the amount of drug measured in the systemic fluids can be converted into a clearance equation by dividing the amount differential equation by a single volume of distribution (the volume of distribution corresponding to the iv bolus dose administered divided by the concentration at time zero, which for KL25A is the dose administered divided by the sum of the coefficients for the iv bolus dose equation, $\frac{2500}{15+16}=80.6\;L$). But then, when deriving the oral dosing equation using mass balance differential equations, what single volume term should be used to convert drug amounts in the systemic circulation to concentrations? The assumed logical conclusion since the beginning of pharmacokinetics was to use the same volume as for the iv bolus dose since one is measuring drug concentrations in the systemic circulation. However, inherent in this choice is the belief that drug input has no effect on *AUC* and that clearance of drug following alternate input processes has no effect on measures of total clearance and is independent of clearance from the delivery site, no matter how slow that process. So, for the past century, our field has concluded for flip-flop models that no matter the terminal rate of elimination determined from the concentration-time curve, clearance is unchanged. But, as we have repeatedly stated in our publications, until we discovered in 2022 that we could derive clearance equations independent of differential equations, it was not possible to derive Equation (21) showing that clearance following alternate input routes could affect the clearance measure depending on the ratio of $\frac{{CL}_{system\;iv\;bolus}}{{CL}_{absorption\;site}}$ and that ${CL}_{absorption\;site}$ could be simply considered as the product of the rate constant for input (i.e., *k_absorption_*) multiplied by the volume of the input site (i.e., *V_absorption site_*), rather than the volume term used to convert the amount differential equation for an iv bolus dose to a concentration relationship. Until now, systemic bioavailability measures greater than unity were not considered possible, with investigators assuming either that absorption led to saturation processes or that experimental errors had to have occurred, in spite of the multiple peer reviewed publications both in animals and humans reporting *F_systemic_* > 1.0.

## 7. Conclusions

All relevant clearance and total rate constant equations in drug metabolism and pharmacokinetics can be simply derived independent of differential equations, based on understanding parallel and in-series rate-defining processes that include all potential drivers, including organ blood flow, net transporter parameters, and drug delivery kinetics. There are no mechanistic implications related to the specific organs of elimination, which is consistent with the clinical reality that only systemic concentrations and urine amounts can be measured. Although our novel approach is controversial as it contradicts certain basic drug metabolism and pharmacokinetic concepts assumed for more than a century, we are unaware of any evidence demonstrating that drug metabolism and clinical pharmacokinetic data are incorrectly analyzed by the approach proposed here. In contrast, there are considerable clinical data, as detailed above, that can only be explained by the approach proposed here.

## Figures and Tables

**Table 1:** Pharmacokinetic information describing iv bolus (Figure 1) and oral (Figure 2) dosing of KL25A to a healthy subject.

IV Bolus		Oral
2500	Dose (mg)	5000
$C_{p}=15\cdot e^{-1.39t}+16\cdot e^{-0.173t}$	Plasma Concentration (µg/mL)—Time (h) Equation	$C_{p}=22.7\cdot e^{-0.105t}-22.7\cdot e^{-1.16t}$
1430	*U_∞_*—Parent Drug Excreted Unchanged in Urine (mg)	220
24	*U_∞,met_*—Drug Metabolites Excreted in Urine (mg)	4
0.10	*f_uB_*	0.10
120	GFR (mL/min)	120
0.9	B/P Blood to Plasma Ratio	0.9

## Data Availability

The original contributions presented in this study are included in the article. Further inquiries can be directed to the corresponding author.

## Abbreviations

The following abbreviations are used in the manuscript:

AUC	Area under the systemic concentration–time curve
AUMC	Area under the moment curve
B/P	Blood to plasma concentration ratio
C_Blood_	Drug concentration in blood
C_in,u_	Unbound concentration of drug in blood entering the liver
C_out,u_	Unbound concentration of drug in blood leaving the liver
C_P_	Drug concentration in plasma CL Clearance
CL_absorption site_	Clearance of drug from the absorption site
CL_bil_	Clearance of drug by biliary excretion
CL_entering_	Entering clearance
CL_filtration_	Renal filtration clearance
CL_H_	Hepatic clearance
CL_H,efflux_	Hepatobasolateral membrane efflux clearance
CL_H,influx_	Hepatobasolateral membrane influx clearance
CL_int_	Intrinsic clearance
CL_leaving_	Leaving clearance
CL_met_	Clearance of drug by hepatic metabolism
CL_R_	Renal clearance
CL_R,reab_	Renal tubular reabsorption clearance
CL_R,sec_	Renal tubular secretion clearance
CL_system iv bolus_	Systemic clearance measured following an iv bolus dose
CL_system oral bolus_	Systemic clearance measured following an oral dose
DM	Dispersion model of hepatic elimination
ER	Extended release
F	Bioavailability
F_Abs_	Fractional bioavailability due to absorption
F_G_	First pass fractional gastrointestinal bioavailability
F_H_	First pass fractional hepatic bioavailability
F_plasma data_	Bioavailability calculated using systemic plasma concentrations
f_uB_	Fraction unbound in the blood
f_uP_	Fraction unbound in the plasma
F_urine data_	Bioavailability calculated based on amounts of unchanged drug in urine
GFR	Glomerular filtration rate
IPRL	Isolated perfused rat liver
IR	Immediate release
IVIVE	In vitro-in vivo extrapolation
k_absorption_	First-order absorption rate constant
k_system iv bolus_	Single rate constant characterizing systemic elimination iv bolus dose
k_system oral_	Single rate constant characterizing systemic elimination oral dose
LDL	Low density lipoprotein
MAT	Mean absorption time
MRT	Mean residence time
PTM	Parallel tube model of hepatic elimination
Q_H_	Hepatic blood flow;
Q_R_	Renal blood flow
T	Time
U_∞_	Total amount of unchanged drug excreted in the urine
U_∞,met_	Total amount of drug metabolites excreted in the urine
V_absorption site_	Volume of distribution of drug of the absorption site
V_ss_	Volume of distribution steady-state
WSM	Well stirred model of hepatic elimination

## References

[1] Wilhemy L.F. (1850). Üeber das gesetz, nach welchem die einwirkung der säuren auf den rohrzucker stattfindet. Ann. Physik Chemie.

[2] Harcourt A.V. (1867). XLIV.—On the observation of the course of chemical change. J. Chem. Soc..

[3] Michaelis L., Menten M. (1913). Die kinetik der invertinwirkung. Biochem. Zeit..

[4] Briggs G.E., Haldane J.B. (1925). A note on the kinetics of enzyme action. Biochem. J..

[5] Pachter J.A., Dill K.A., Sodhi J.K., Benet L.Z. (2022). Review of the application of Kirchhoff’s Laws of series and parallel flows to pharmacology: Defining organ clearance. Pharmacol. Ther..

[6] Benet L.Z., Sodhi J.K., Tiitto M.V., Xiang Y. (2025). Application of Kirchhoff’s Laws to pharmacologic and pharmacokinetic analyses. Pharmacol. Rev..

[7] Benet L.Z., Sodhi J.K. (2025). Simplifying pharmacokinetics, applying it to drug and dosage form development, and making drug dosage decisions in clinical medicine: The adaptation of Kirchhoff’s Laws from physics. AAPS J..

[8] Kirchhoff G. (1857). Ueber die bewegung der electricität in leitern. Ann. Phys..

[9] Kirchhoff G. (1857). Ueber die bewegung der electricität in drähten. Ann. Phys..

[10] Yamaoka K., Nakagawa T., Uno T. (1979). Statistical moments in pharmacokinetics. J. Pharmacokinet. Biopharm..

[11] Cleland W.W. (1975). Partition analysis and the concept of net rate constants as tools in enzyme kinetics. Biochemistry.

[12] Benet L.Z. (1972). General treatment of linear mammillary models with elimination from any compartment as used in pharmacokinetics. J. Pharm. Sci..

[13] Benet L.Z., Sodhi J.K. (2023). The uses and advantages of Kirchhoff’s Laws vs. differential equations in pharmacology, pharmacokinetics, and (even) chemistry. AAPS J..

[14] Benet L.Z., Sodhi J.K. (2026). Net influx rather than directional rates: Re-evaluating transporter characterization in vivo and in vitro for renal and hepatic clearance. AAPS J..

[15] Garrison K.L., Sahin S., Benet L.Z. (2015). Few drugs display flip-flop pharmacokinetics and these are primarily associated with classes 3 and 4 of the BDDCS. J. Pharm. Sci..

[16] Bricker N.S., Morrin P.A., Kime S.W. (1960). The pathologic physiology of chronic Bright’s disease. An exposition of the “intact nephron hypothesis”. Am. J. Med..

[17] Rowland M., Benet L.Z., Graham G.G. (1973). Clearance concepts in pharmacokinetics. J. Pharmacokinet. Biopharm..

[18] Wilkinson G.R., Shand D.G. (1975). Commentary: A physiological approach to hepatic drug clearance. Clin. Pharmacol. Ther..

[19] Sodhi J.K., Wang J.H., Benet L.Z. (2020). Are there any experimental perfusion data that preferentially support the dispersion and parallel-tube models over the well-stirred model of organ elimination?. Drug Metab. Dispos..

[20] Keiding S., Chiarantini E. (1978). Effect of sinusoidal perfusion on galactose elimination kinetics in perfused rat liver. J. Pharmacol. Exp. Ther..

[21] Benet L.Z., Liu S., Wolfe A.R. (2018). The universally unrecognized assumption in predicting drug clearance and organ extraction ratio. Clin. Pharmacol. Ther..

[22] Li X., Jusko W.J. (2022). Assessing liver-to-plasma partition coefficients and in silico calculation methods: When does the hepatic model matter in PBPK?. Drug Metab. Dispos..

[23] Sirianni G.L., Pang K.S. (1997). Organ clearance concepts, new perspectives on old principles. J. Pharmacokinet. Biopharm..

[24] Benet L.Z., Bowman C.M., Liu S., Sodhi J.K. (2018). The extended clearance concept following oral and intravenous dosing: Theory and critical analyses. Pharm. Res..

[25] Benet L.Z., Sodhi J.K. (2025). Response to the commentary on “Are all measures of liver Kp_uu_ a function of *F_H_*, as determined following oral dosing, or have we made a critical error in defining hepatic clearance?”. Eur. J. Pharm. Sci..

[26] Benet L.Z., Sodhi J.K. (2024). Pharmacokinetic theory must consider published experimental data. Drug Metab. Dispos..

[27] Benet L.Z., Sodhi J.K. (2024). Are all measures of liver *Kp_uu_* a function of *F_H_*, as determined following oral dosing, or have we made a critical error in defining hepatic clearance?. Eur. J. Pharm. Sci..

[28] Benet L.Z., Galeazzi R.L. (1979). Noncompartmental determination of the steady-state volume of distribution. J. Pharm. Sci..

[29] Lukacsko P., Walters E.J., Cullen E.I., Niecestro R., Friedhoff L.T. (2004). Efficacy of once-daily extended-release lovastatin as compared to immediate-release lovastatin in patients with hypercholesterolemia. Curr. Med. Res. Opin..

[30] Davidson M.H., Lukacsko P., Sun J.X., Phillips G., Walters E., Sterman A., Niecestro R., Friedhoff L. (2002). A multiple-dose pharmacodynamic, safety, and pharmacokinetic comparison of extended and immediate-release formulations of lovastatin. Clin. Ther..

[31] Benet L.Z., Tiitto M.V., Sodhi J.K. (2024). The potential of developing high hepatic clearance drugs via controlled release: Lessons from Kirchhoff’s Laws. J. Control. Release.

[32] Wakuda H., Xiang Y., Sodhi J.K., Uemura N., Benet L.Z. (2024). An explanation of why dose-corrected area under the curve for alternate administration routes can be greater than for intravenous dosing. AAPS J..

